# A standardized method for vertical sleeve gastrectomy bariatric surgery investigations in cancer

**DOI:** 10.3389/frmbi.2025.1432817

**Published:** 2025-07-15

**Authors:** Arvind V. Ramesh, Sydney C. Joseph, Margaret S. Bohm, Emily W. Grey, Joel H. Elasy, Brianne M. Hibl, Oluwatosin T. Asunloye, Ki-Suk Kim, Teri D. Doss, Joseph F. Pierre, Katherine L. Cook, Liza Makowski, Laura M. Sipe

**Affiliations:** ^1^ Division of Hematology and Oncology, Department of Medicine, College of Medicine, University of Tennessee Health Science Center (UTHSC), Memphis, TN, United States; ^2^ Department of Microbiology, Immunology, and Biochemistry, College of Medicine, UTHSC, Memphis, TN, United States; ^3^ Office of Comparative Medicine, University of Utah, Salt Lake City, UT, United States; ^4^ Department of Physiology, College of Medicine, UTHSC, Memphis, TN, United States; ^5^ Department of Molecular Physiology and Biophysics, Vanderbilt Mouse Metabolic Phenotyping Center, Vanderbilt University, Nashville, TN, United States; ^6^ Department of Nutritional Sciences, College of Agriculture and Life Science, The University of Wisconsin-Madison, Madison, WI, United States; ^7^ Department of Cancer Biology, Wake Forest University School of Medicine, Winston-Salem, NC, United States; ^8^ UTHSC Center for Cancer Research, College of Medicine, UTHSC, Memphis, TN, United States; ^9^ Department of Biology, University of Mary Washington, Fredericksburg, VA, United States

**Keywords:** obesity, breast cancer, Peyer’s patch, anti-tumor immunity, microbiota

## Abstract

Obesity is a global epidemic that has affected the lives of over 14% of adults worldwide and over a third of Americans. Obesity is associated with the increased risk of thirteen obesity-associated cancers and poor cancer outcomes. Bariatric surgery is the most effective method of sustained weight loss and has been steadily increasing in clinical use over the past 4 decades. Importantly, bariatric surgery is established to decrease cancer risk. Vertical sleeve gastrectomy (VSG) is currently the most common bariatric surgery procedure. To evaluate underlying mechanisms of bariatric associated cancer protection, we developed a robust pre-clinical model of bariatric surgery-induced weight loss in mice. Using multiple strains, we established detailed procedures, defined best practices, and noted specific controls to include to examine mediators critical to cancer onset. This VSG protocol includes stringent pre- and post-operational measures to reduce stress-associated weight loss in obese mice to achieve rigorous and reproducible bariatric surgery-associated weight loss. In addition, we describe collection of fecal and intestinal samples as well as Peyer’s patches as important mediators of bariatric surgery’s impact on cancer risk. In conclusion, as obesity and weight loss approaches including bariatric surgery are increasingly examined in cancer risk and outcomes including immunotherapy, the establishment of robust pre-clinical interventions will allow the field to address critical underlying mechanisms mediating the benefits of weight loss and cancer.

## 1 Introduction

Obesity, defined as having a body mass index (BMI) at or above 30 kg/m^2^, is a pressing epidemic that is increasingly impacting countries around the world. As of 2023, the worldwide adult obesity prevalence was 14% (World Obesity, 2023). The mean BMI of both adult males and females has steadily increased since the first reports of an obesity epidemic in 1975 (Collaboration, 2016; Valenzuela et al., 2023). Current projections suggest that the obesity rate will continue to rise to a projected 25% by 2035 (World Obesity, 2023). Obesity is associated with metabolic syndrome, a condition associated with the development of cardiovascular diseases, cerebrovascular diseases, and type 2 diabetes, highlighting the complex disease mechanisms involved in obesity-associated pathologies (Kunes et al., 2023). In addition, obesity is associated with increased risk of thirteen cancers as defined by the International Agency for Research on Cancer (IARC) Working Group and is implicated in poorer outcomes and increased mortality (National Cancer Institute, 2022). According to the World Health Organization (WHO), obesity results in approximately 2.8 million deaths each year, indicating a vital need for therapeutics and interventions targeting the obesity pandemic (World Health Organization, 2021).

Despite a variety of therapeutic options seeking to reduce obesity, including lifestyle interventions such as physical activity, specific diets, pharmaceuticals, and surgery, obesity rates are rising. When lifestyle interventions or other treatments fail, bariatric surgery is a viable option for some patients, as reviewed in Bohm et al. (2022). In those categorized with severe obesity, a BMI > 40kg/m^2^ or > 35 kg/m^2^ with at least one obesity-associated comorbidity, bariatric surgery is highly effective (Altieri et al., 2021; Bohm et al., 2022; Liu and Funk, 2020; Ruban et al., 2019). Bariatric surgery produces the greatest weight loss compared to non-surgical methods (Muller et al., 2022; Ruban et al., 2019) and significantly improves obesity-associated metabolic dysfunction (Bjornstad et al., 2020; Buchwald et al., 2014; Derderian et al., 2020; Mollan et al., 2021; Purnell et al., 2021; Sjostrom et al., 2007). Various bariatric surgery procedures have been implemented historically with accompanying mouse models developed to examine impacts on physiology (Amouyal et al., 2020; Liou et al., 2013). Currently, vertical sleeve gastrectomy (VSG) is the most common type of weight loss surgery performed, outpacing Roux-en-Y gastric bypass (RYGB), with gastric banding falling out of favor in the past decade (Lewis et al., 2021). VSG is primarily performed via laparoscopic methods with very low rates of side effects (Buchwald et al., 2004; Liu and Funk, 2020; Nguyen et al., 2004; Varela and Nguyen, 2015). RYGB also has excellent long-lasting weight loss benefits and similar cancer-risk reduction benefits, but RYGB is a more invasive surgery that involves a restrictive surgical intervention combined with rearrangement of the intestines (Arterburn et al., 2020; Ayer et al., 2017). VSG solely consists of removing the greater curvature of the stomach, including the fundus, to restrict food intake (Melissas et al., 2008). VSG is also accompanied by increased gastric emptying and hormonal changes (Arterburn et al., 2020; Evers et al., 2017; Varela-Rodriguez et al., 2020). Consequently, this methods manuscript provides an updated VSG protocol to study obesity, weight loss, and potential mediators of cancer onset and progression. The weight loss, intestinal sampling, and immunity approaches detailed herein may be applied to any obesity associated disease or any cancer. Our group has published on mainly breast cancer, pancreatic cancer and lung cancer (Bohm et al., 2025; Marathe et al., 2025; Pingili et al., 2021; Sipe et al., 2022). However, there is elevated risk of over thirteen cancers associated with obesity noted by the International Agency for Research on Cancer (IARC) including postmenopausal breast cancer, colorectal cancer, endometrial/uterine cancer, esophageal adenocarcinoma, gall bladder cancer, gastric cancer, hepatocellular cancer, meningioma, multiple myeloma, ovarian cancer, pancreatic cancer, renal cancer, and thyroid cancer (Clinton et al., 2020; Lam et al., 2024; Lauby-Secretan et al., 2016).

Taken together, clinical and epidemiologic data support a positive role of weight loss in protection from a variety of serious diseases, but the specific mechanisms underlying this protection remain uncertain. Therefore, modeling bariatric surgery in the preclinical setting provides vital insight into the mechanisms of surgical weight loss-induced disease protection. Bariatric surgery is difficult to perform in mice due to the need for high precision and careful pre- and postoperative care to reduce weight loss due to stress. While most mice examined in obesity and bariatric surgery studies are C57BL/6, this strain is the least used in cancer models. Therefore, the use of bariatric surgery in different laboratory mice strains is discussed regarding strain-specific differences in response to the surgery. Female mice are not well represented in literature regarding bariatric surgery. Herein, we detail a VSG procedure, including both preoperative and postoperative procedures, on obese female mice. This procedure has been successful in both demonstrating the role of VSG and the gut microbiome through FMT in obesity and breast cancer studies, including response to immunotherapy (Bohm et al., 2025, 2024; Sipe et al., 2022). We also describe the collection of the gut microbiota and immune associated cells in the Peyer’s patches of the intestines as important mediators of local and systemic effects of bariatric surgery. Taken together, this highly detailed description of the VSG method, devised after years of training with leaders in the field, workshops, hands on and observational practice, combined with technical training tables with up to date resources, examples of data, and citations to support the validation of this method, this manuscript shall serve as a reliable resource for a starting point to increase the study of bariatric surgery in obesity associated pathologies from cancer to diabetes to atherosclerosis and beyond.

## 2 Vertical sleeve gastrectomy protocol

Before performing any surgeries, consult with the members of your Institutional Animal Care and Use Committee (IACUC) and veterinary staff to ensure the best outcomes. One should optimize aseptic techniques and suturing skills before learning to perform the surgery. Ensure that there is an adequate procedure room for two staff members, necessary anesthesia equipment, and cautery tools. There should be enough space for a dissecting scope to perform the surgery such as the SZX7 model (Olympus, Center Valley, PA). All supplies, reagents, and materials noted below are listed in **Table 1**.

**Table 1:** Reagents and materials for bariatric surgery.

Consumables	Catalog Number	Notes
Puritan 6” Sterile Standard Cotton Swab	25-806-10pPC	*10 Pack ideal
USP Type VII Gauze Sponges 2x2in	2252	Autoclaved
Dermacea Non-Woven Sponge, 2 x 2 Inch Square	441403	
Betadine solution (povidone-iodine solution USP, 10%)	NDC 67618-150-01	
Puralube^®^ Ophthalmic Ointment	1000059390	Used to prevent eyes from drying out
Prefilled syringe 0.9% Saline 10ml	91082	
Alcohol Prep Pads	MFR 58-204	
3M™ Steri-Drape™ Small Towel Drape	1000NSD	
Towel Drapes Poly Lined 18”x26”	4410	
Sterile Gloves	MSG5070	
Petri Dish- Sterile	174945	
8–0 COATED VICRYL VIOLET 1X12” TG140–8 DOUBLE ARMED	J548G	
5–0 COATED VICRYL VIOLET 1X18” FS-2	VCP391	
Reflex 7mm Wound Clips 1000 Pack	203-1000	
Bovie cautery low temperature micro fine tip	AA90	
Ensure liquid diet Original nutritional shake Milk Chocolate	53623	
Nair		Commercially purchased
Surgical Tools	Catalog Number	Notes
Reflex 7mm Wound Clip Applier	RS-9250	
Micro Mosquito Forceps- Roboz Surgical Store	RS-7117	Used to clamp the stomach
Surgical Scissors		
5” Bracken Curved Forceps		
4” Bracken Curved Forceps		
Spring Scissors		
Needle Drivers/Holder		
Micro Needle Drivers		Recommend 5+ inches long

This table lists consumables and surgical tools necessary to conduct bariatric surgery with notes if necessary.

Study design, choice of mouse model, and approach to diet-induced obesity (DIO) should be carefully considered in consultation with experts in obesity and metabolism (Glenny et al., 2021). Mice should be age and sex matched for experimental and control groups. Mice should be purchased with enough time to allow for 2 weeks of acclimation before studies initiate. Mice may be purchased from companies such as The Jackson Labs (JAX) already obese on the diet of choice as arranged with JAX or made obese in house. Diet induced obesity has been reviewed extensively by our group and others (Bohm et al., 2022; Sipe et al., 2020). Briefly, lard based diets are frequently used such as the DIO series sold by Research Diets Inc, however, dietary pattern style diets (Mediterranean, Western, Cafeteria Diet), or diets to test specific fats or other macronutrients are options as standard or custom diets (Arnone et al., 2024; Johnson et al., 2016; Sampey et al., 2012, 2011). A main concern in dietary and DIO study design is to always use defined controls to ensure matching on micronutrients (vitamins and minerals), fiber, compounds such as phytoestrogens, and keep status on irradiation or not consistent (the latter often depends on the rules of the animal facility) (Engber, 2018). Mice should be randomized and housed to match on initial starting weights. After a baseline weight and body composition by EchoMRI if possible, weights should be measured at least weekly (Pingili et al., 2021). DIO may be defined by a percentage weight gain depending on the age of mice when the diet was initiated. For example, in our hands, female mice weaned onto 60% high fat diet more than doubled body weight after 16 weeks on diet with fat mass at 15-17% before bariatric surgery compared to lean mice with 3-4% fat mass in lean mice on low fat diet (Sipe et al., 2022). Some mice may be resistant to DIO and can be excluded by a rule of thumb reported in the methods section (such as mice weighing less than 28 g after 16 weeks on 60% lard based high fat diet (Sipe et al., 2022)). Lastly, care should be taken when incorporating immune and microbiome endpoints in cancer studies, as defined in this method manuscript, which was recently reported in detail by our team (Bohm et al., 2025).

### 2.1 Pre-operative preparation for surgical instruments and mouse diet

**Figure 1:**
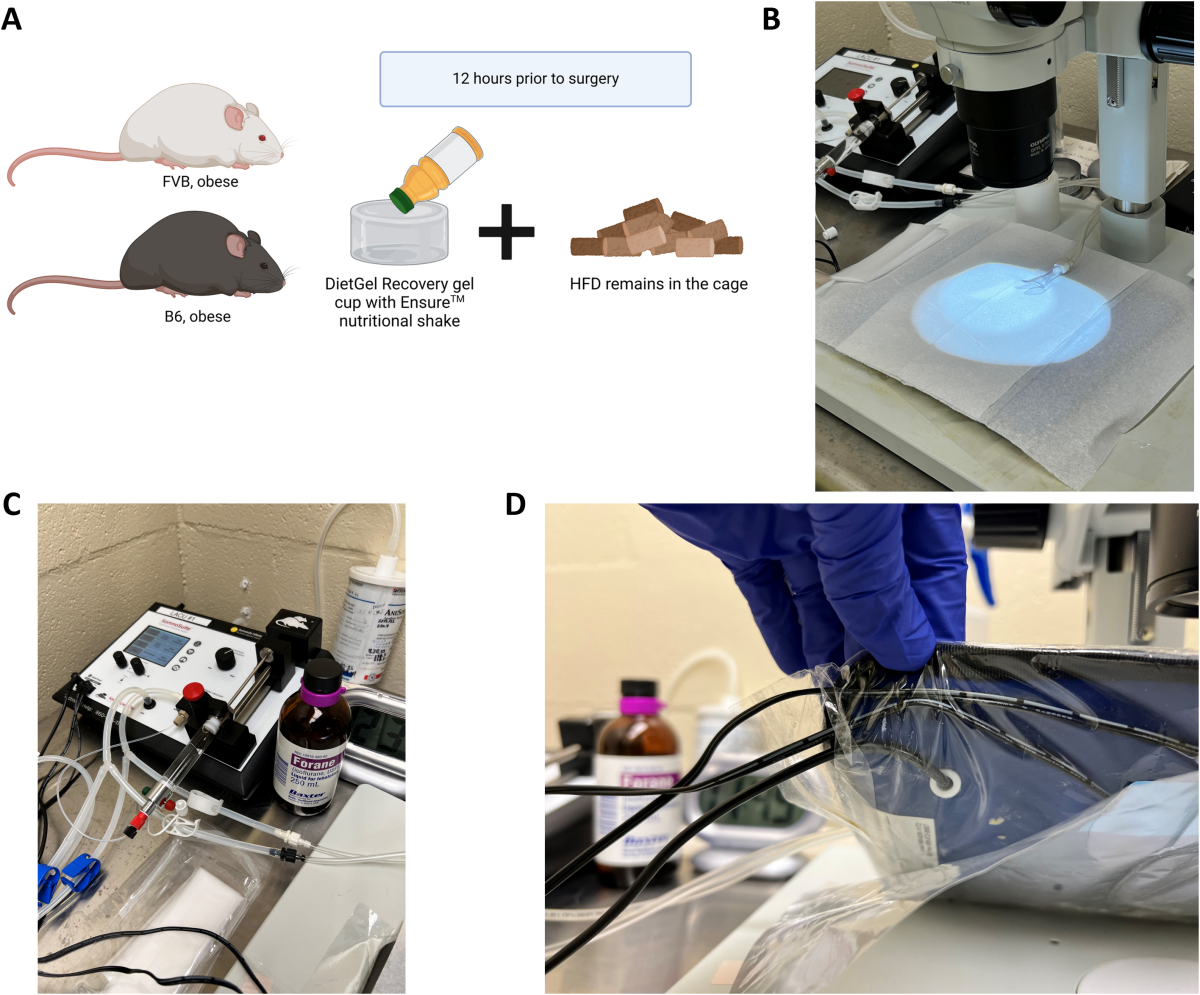
Pre-surgical preparation and anesthesia set up. **(A)** Diet induced obese or genetically obese murine models such as the C57BL/6J (“B6”, highly obesogenic) or the FVB/N (“FVB”, moderately obesogenic) may be prepared the day before the surgery by adding DietGel and Ensure nutritional shake along with high fat diet (HFD) pellets to being to reduce food content in the stomach. **(B)** The procedure table should be cleaned, disinfected and the surgical field draped with a sterile towel under the dissecting microscope. **(C)** An anesthesia apparatus such as the SomnoSuite Low-Flow Anesthesia System should be nearby and prepared for surgery with anesthesia such as isoflurane. **(D)** The heating pad associated with the SomnoSuite and under the sterile pad will be turned on. The heating pad sensors underneath the pad itself should be secured with masking or surgical tape to avoid movement during surgery.

1. Autoclave all surgical instruments and non-sterile gauze, at 121.9°C for 30 minutes at 14.47 psi.
2. 12 hours before surgery: introduce mice to 1 cup of DietGel Recovery (2 oz., 72-06-5022, Clear H_2_O, Westbrook, ME) with about 0.35 oz of Ensure™ Original Nutrition Shake (53623, Abbott Laboratories, Columbus, OH) poured on top of the gel in addition to their normal solid food pellets, typically high fat diet (HFD, cartoon **Figure 1A**), to mimic a liquid surgical diet.
3. 4 hours prior to surgery: to reduce food bulk in the stomach, place mice into a new clean cage, which reduces risk of infection and odor over time. Remove solid food pellets, leaving the DietGel Recovery with Ensure liquid diet poured on top of the gel in the cage.

### 2.2 Procedure room preparations

1. Disinfect the tabletop where the surgery will be performed with a facility-approved disinfectant (such as Peroxigard Ready-to-Use One Step Disinfectant Cleaner & Deodorizer or 70% ethanol).
2. Place a sterile towel drape (poly-lined, 18”x 26”) on the surgical field (**Figure 1B**).
3. Prepare your anesthetic machine as necessary for your specific system (fill isoflurane, check supply gas levels, etc.). We recommend using a SomnoSuite Low-Flow Anesthesia System (Kent Scientific, Torrington, CT, **Figure 1C**) set up with 5mL of isoflurane.
4. Ensure a heating source is placed on the surgical surface under the dissecting scope (SZX7, Olympus, Center Valley, PA). Our recommendation is to use the Far Infrared Warming Pad that is incorporated into the SomnoSuite via the RightTemp^®^ system. This system will control the temperature of the heated surface based on the animal’s temperature and the maximum allowed temperature of the pad itself. To set up the RightTemp^®^ system, tape the heating pad sensors (shown in **Figure 1D**) underneath the pad itself to avoid movement during surgery with masking or surgical tape.
5. Cover the heating pad with 1–2 dry paper towels and tape down to secure.
6. Place a syringe containing 0.9% Sodium Chloride Injection, USP (30654, Becton, Dickinson and Company, Franklin Lakes, NJ) beneath a second heating pad (Kent Scientific, Torrington, CT) set to 43°C to warm it up. If available, an incubator can be used to pre-heat a bottle or bag of fluids as opposed to a syringe of fluids being heated via a heating pad.

### 2.3 Animal pre-operative preparation

**Figure 2:**
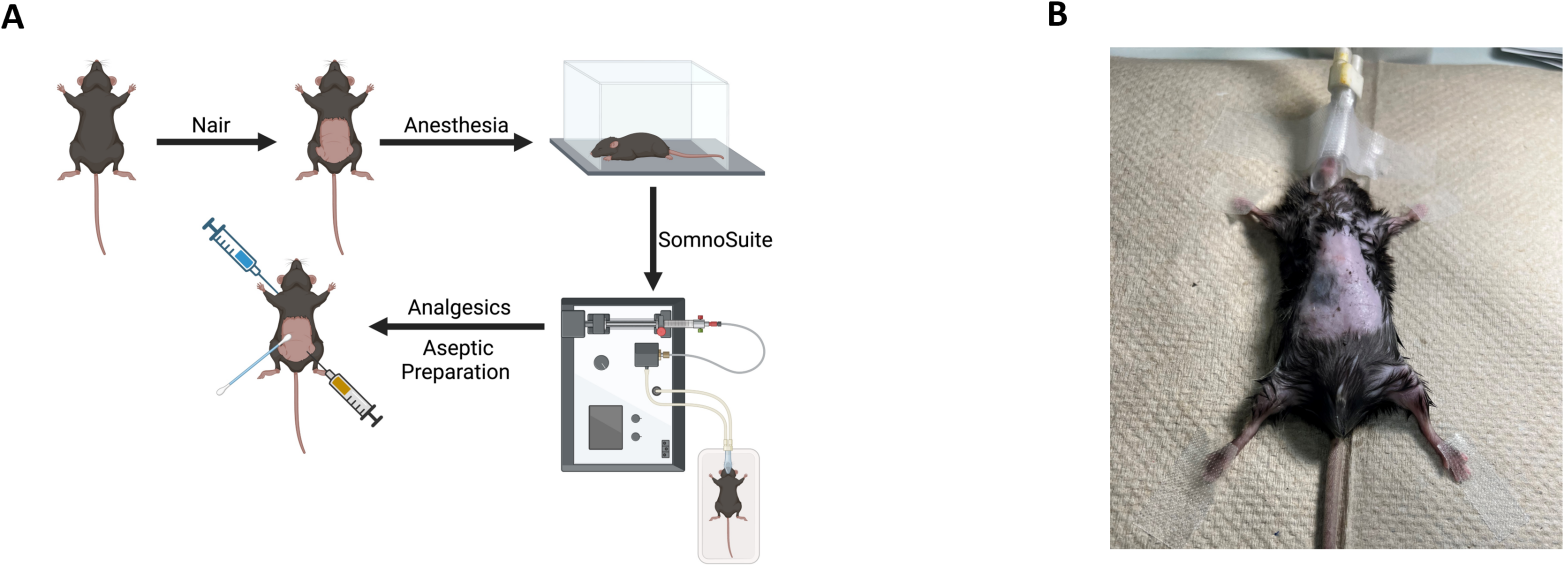
Pre-surgical preparation of the mouse. **(A)** Schema demonstrating the use of Nair™ to remove hair from the surgical site. Use of the SomnoSuite induction chamber, nose cone, and sterilization of the surgical site by betadine antiseptic solution is shown. The percentage and flow of isoflurane will vary based on the weight of the mouse. **(B)** Once the mouse is properly anesthetized in the induction chamber, transfer the mouse to the anesthesia nose cone of the SomnoSuite. Lay the mouse in a supine position on the prepared surgical field. The nose cone and the paws of the mouse should be taped down to avoid potential movement.

**Figure 3:**
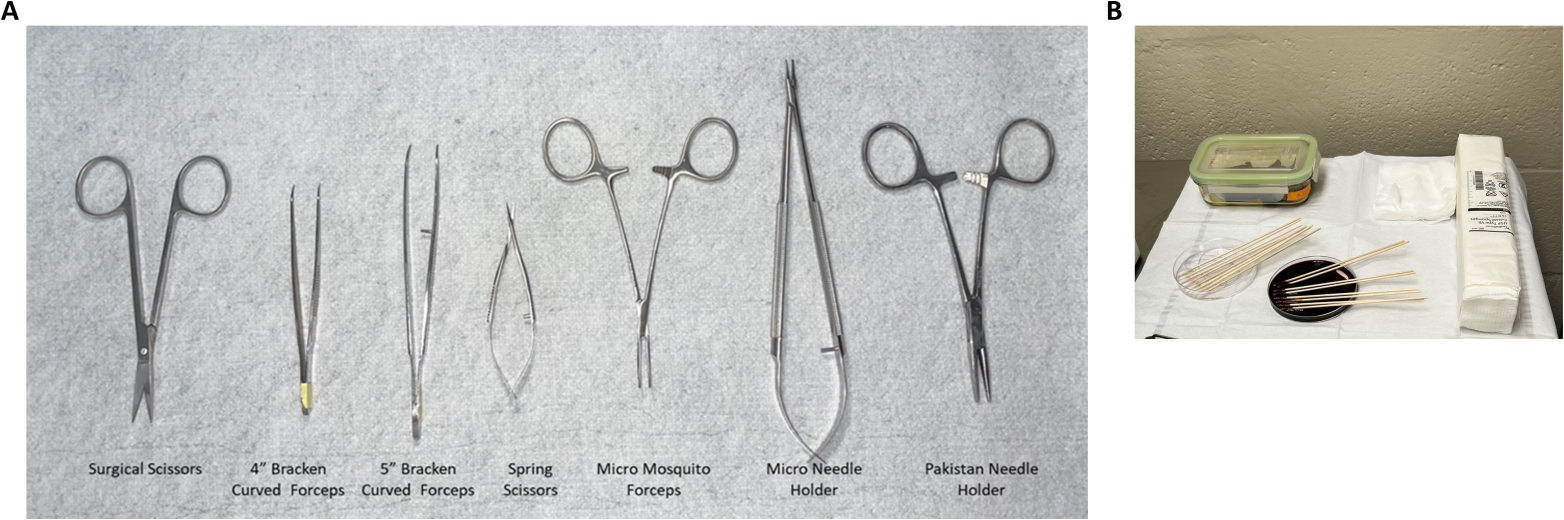
Surgical tool preparation and procedure table set up. **(A)** Various scissors, forceps, and holders are shown with labels across the bottom that are described in this protocol. **(B)** Lay out autoclaved surgical tools and supplies such as gauze and cotton tip applicators on the sterile drape adjacent to the surgical field.

1. Determine surgical site. The incision should be 2–3 cm long and in the center of the abdomen.
2. **Figure 2A** is a cartoon of the pre-operative preparation and anesthesia. Remove hair from the surgical site by applying Nair™ (non-fragrant, Church & Dwight Co., Inc., purchased from local pharmacy) to the surgical area of the mouse for 30 seconds. Remove with a warm water-soaked gauze within 30 seconds to avoid skin irritation. Repeat step if necessary to remove all hair on the surgical site (**Figure 2B**). Another option is that mice can be shaved if preferred, but Nair is better at removing all traces of hair.
3. Place mouse in the SomnoSuite induction chamber and begin anesthetic exposure using about 3.0%-5% isoflurane.
4. Once the mouse is properly anesthetized, transfer the mouse from the induction chamber to the anesthesia nose cone of the SomnoSuite.
5. Perform a subcutaneous (SQ) injection in the shoulder area of a non-steroidal anti-inflammatory drug (NSAID)at a dose appropriate for a mouse. We utilized Carprofen (#141-199, Zoetis, Parsippany-Troy Hills, NJ) 5–10 mg/kg. Consult your veterinary staff for carprofen dosing to avoid stomach irritation or bleeding. NSAIDS are the preferred pain control medication because the use of opioids may slow intestinal motility and ideally should be avoided during bariatric surgery (**Table 2**). Then lay the mouse in a supine position on the prepared surgical field.
6. Ensure the mouse’s nose is sitting firmly within the nose cone (**Figure 2B**).
7. Using masking or surgical tape (#16-47220, McKesson, Irving, TX), tape the nose cone to the table to avoid nose cone displacement during surgery (**Figure 2B**).
8. Last, tape the front paws of the mouse spread to fully expose the abdomen, but not too tight to allow the animal to breathe freely (**Figure 2B**). Ensure the mouse is fully anesthetized by doing a toe-pinch test. Successful anesthetization of the mouse will result in no leg flexion during the toe-pinch test.
9. Supply approximately 49 mL/min of isoflurane at 2%-3% to a 40 g mouse. The SomnoSuite automatically adjusts flow based on weight of mouse and will vary if low flow or standard anesthetic machine.
10. Decide on analgesics (single or combination) with the veterinary consultation at your university. We perform an intradermal (ID) injection at the incision site using 50 µL of a lidocaine-bupivacaine mix (1:1 mix of 2% lidocaine and 0.5% bupivacaine diluted 1:20 in medical grade saline), making 3–4 small injections along the 2–3 cm incision area. Bupivacaine alone or liposomal bupivacaine are also options. Lidocaine (#11695-4149-1, Covetrus, Portland, ME) and bupivacaine (Gifted by UTHSC Laboratory Animal Care Unit) are non-DEA regulated pain medications (**Table 2**).
11. Aseptically prepare the shaved area of the mouse’s abdomen as appropriate. We recommend preparation with cotton tip applicators soaked in betadine antiseptic solution (Povidone-Iodine solution USP, 10%, (NDC67618-150-01, Purdue Products L.P., Stamford, CT) which sits for a minimum of 15 seconds prior to being wiped with an alcohol prep pad and repeating this process three times.
12. Cut a small 3M™ Steri-Drape™ (3M Health Care, Neuss, DE) into thirds. Place one third over the mouse’s abdomen and cut a small hole approximately 4 cm to access the incision site.
13. Perform another toe-pinch test to ensure the mouse is fully anesthetized.
14. Change into sterile surgical gloves (MSG5070, Thermo Scientific, Waltham, MA).
15. Prepare autoclaved surgical tools by opening a sterile instrument peel pack containing all of the instruments. Place autoclaved supplies on the sterile drape (**Figures 3A,B** **).**
16. Disinfect the surgical tools in between surgeries using a Micro Bead Sterilizer (6683, Cell Point Scientific, Gaithersburg, MD) set to 260°C. Spray tools with 70% ethanol and thoroughly wipe clean until dry before placing them into the bead sterilizer. Improper wiping of ethanol prior to sterilizing the tools can result in a fire. Place the tools in the bead sterilizer for at least 5 seconds each time.

**Table 2:** Medications used throughout the bariatric VSG procedure.

Agents	Dose	Route	Frequency	Duration
Carprofen	5–10 mg/kg	SQ (shoulders)	24 hours	3 days
Lidocaine/Bupivacaine	50 µL	Intradermal at incision site	At time of surgery	N/A
Buprenorphine*	0.05-0.1 mg/kg	SQ	As needed	N/A

This table details the medications necessary or optional, as denoted by *, for the vertical sleeve gastrectomy (VSG) bariatric surgery. Buprenorphine is cautioned against use because it may slow gastric emptying.

### 2.4 Vertical sleeve gastrectomy surgical procedure and sham surgery control procedure

**Figure 4:**
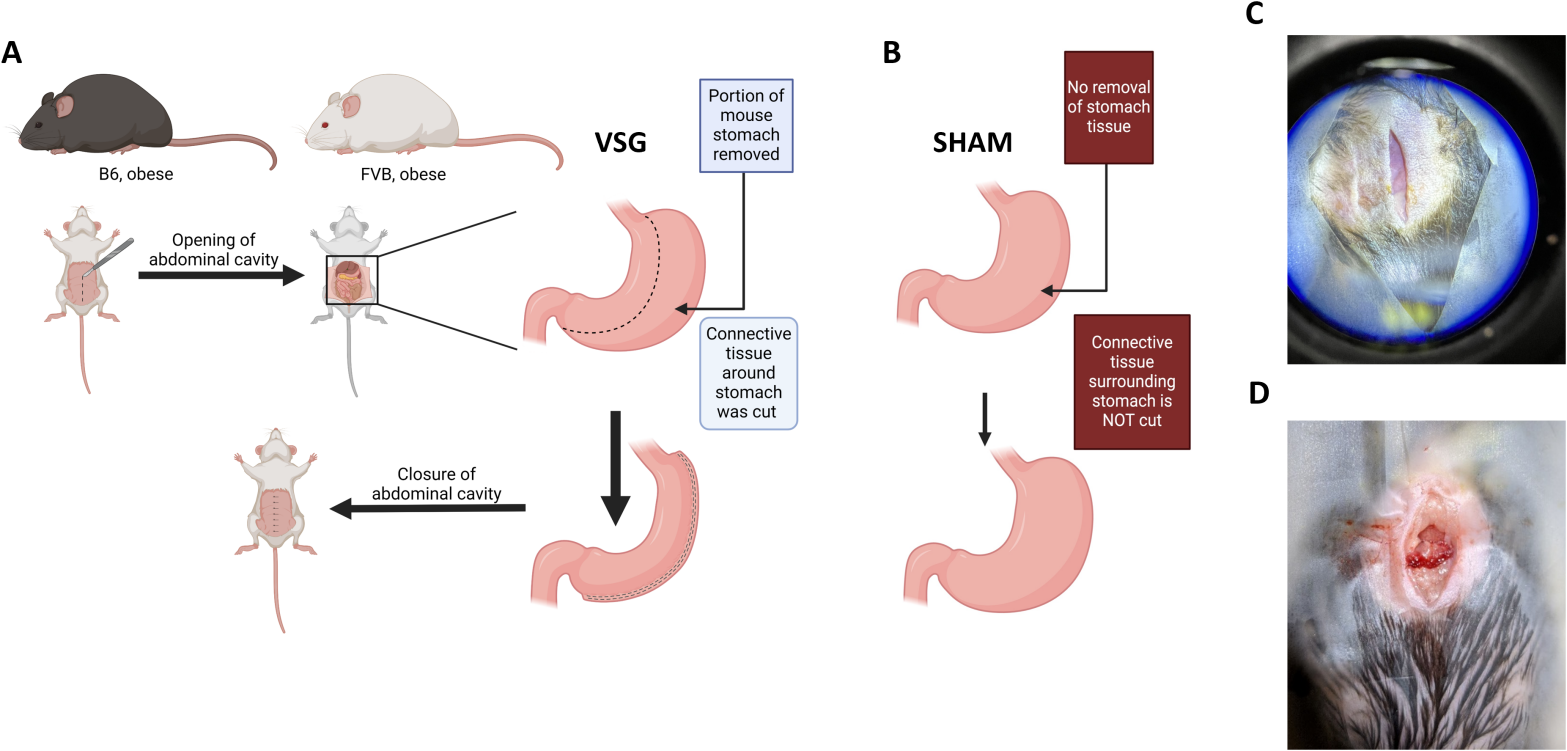
Midline incision of the abdomen for VSG and sham surgery. **(A–B)**. Schema of the opening of the skin then the abdominal cavity to prepare for either **(A)** the VSG removal of a portion of the stomach or **(B)** the sham surgical procedure where the stomach remains intact. **(C)** Incision of the skin by a midline laparotomy should be between 2–3 cms along the linea alba away from the rectus abdominus muscles. **(D)** Incision of the muscle layer will open the peritoneal cavity.

Note: During surgery, continuously monitor the animal’s vital signs (e.g., Respiratory rate), response to noxious stimuli, and spontaneous movement.

**Figure 4A** is a cartoon of the vertical sleeve gastrectomy (VSG) procedure, while **Figure 4B** depicts the control sham surgery procedure. The VSG includes points 1–18 in section A.4, while the sham surgery includes only 1-3 (omitting steps 4-15) and finishing with the same closures as the VSG in steps 16-18.

**Figure 5:**
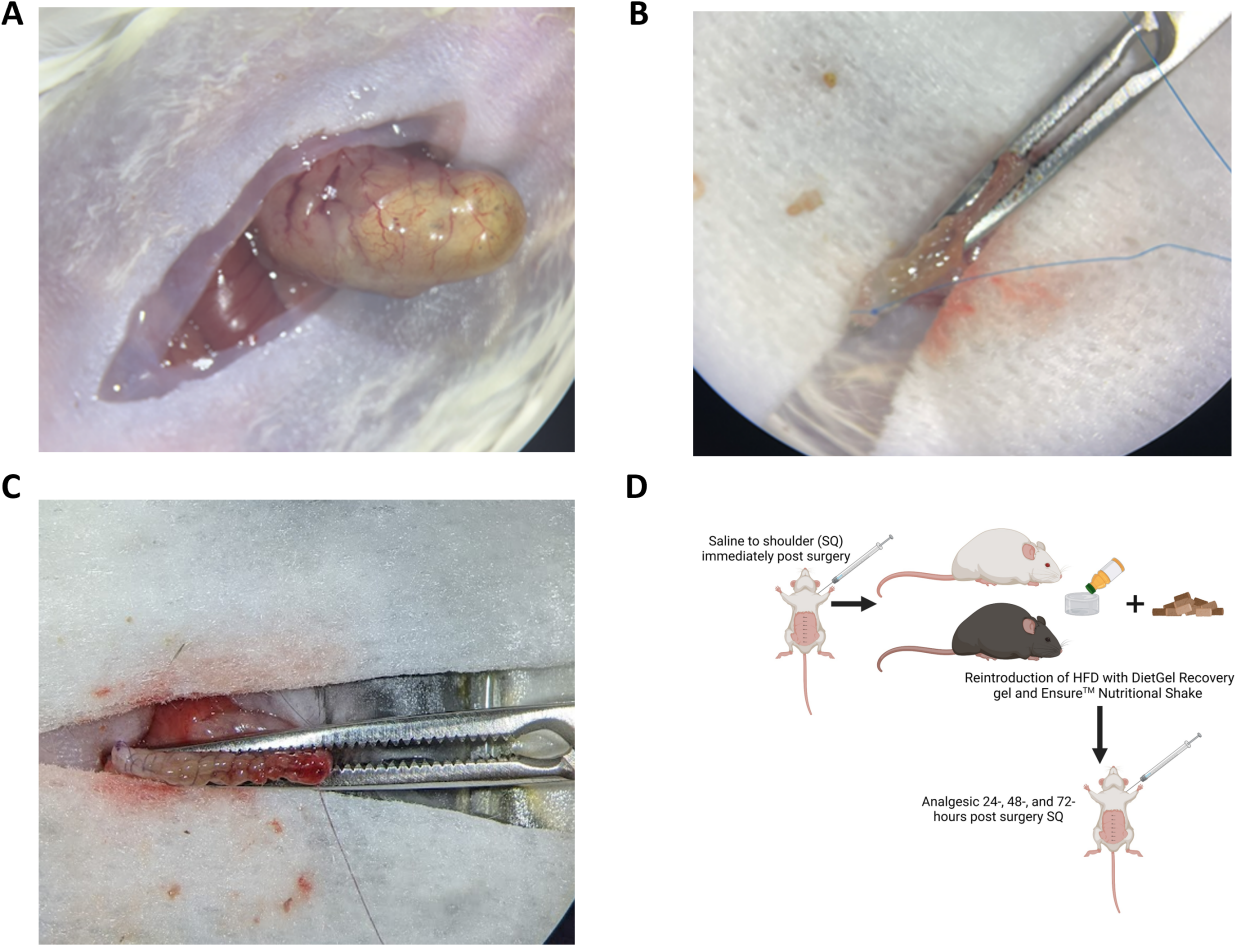
Isolation of stomach. **(A)** Gently lift the stomach out of the peritoneal cavity by cutting away connective tissue, using cautery to reduce bleeding as described in protocol. **(B)** Use a clamp (Micro Mosquito Forceps) from the base of the esophagus to the pancreatic vessel with curved jaws facing down. **(C)** Cut away the greater curvature of the stomach, including the fundus (**Figure 4A**), along the convex side of the clamp, leaving 2 mm of stomach extending from the clamp to allow for suturing to close the stomach. **(D)** Continuous suturing technique should be used along the clamp to close the stomach.

1. First, start with incising the skin. Using sharp surgical scissors, begin to make a midline laparotomy incision. The incision should be 2–3 cm. Ensure that the incision is made along the linea alba and away from the rectus abdominis muscles (**Figure 4C**).
2. Next, incise the muscle layer which will make available the peritoneal cavity (**Figure 4D**).
3. Using sterile, saline (30654, Becton, Dickinson and Company, Franklin Lakes, NJ) soaked cotton tip applicators, gently shift the liver and other abdominal organs out of the way to locate the stomach.
4. Gently dissect the stomach from any abdominal connective tissue using spring scissors. Use care not to cut any of the mesentery attached to the spleen or the pancreas until appropriate arteries have been ligated via cautery. Gently lift the stomach outside of the abdominal cavity (**Figure 5A**).
5. Once the stomach is clear of connective tissue and the peritoneal cavity, place a wet cotton tip applicator behind the exposed splenic artery. Using a micro fine tip low-temperature Bovie cautery (AA90, Bovie, Clearwater, FL), cauterize the splenic artery. Successful cauterization of the vessel will immediately turn the vessel white. After successful cauterization, cut the artery at the cauterization site.
6. Gently pull back the pancreas from the stomach using a wet cotton tip applicator to expose the pancreatic artery. Avoid touching the pancreas as much as possible since it is highly sensitive to damage. Cauterize the pancreatic artery using the Bovie cautery. After successful cauterization, cut the artery at the cauterization site. Place light pressure on the artery with a saline-soaked cotton tip applicator to stop any excessive bleeding, if needed.
7. Cut away remaining connective tissue attaching the stomach to the spleen to further release the stomach.
8. Take a non-woven gauze sponge and make a cut halfway through the gauze sponge. Wet the sponge with sterile 0.9% saline and place behind the stomach to properly hold the stomach in the correct orientation. Ensure that the pancreas and other organs are tucked back into the abdominal cavity leaving the stomach alone outside of the cavity resting on the gauze.
9. Place an additional dry woven gauze under the stomach. Using spring scissors, cut one end of the fundus open. Use a saline soaked cotton swab to roll the stomach contents out and onto the woven gauze.
• Use additional woven gauze to catch all stomach contents.
• Great care should be taken to ensure no stomach contents fall into the abdominal cavity and are instead caught by the dry woven gauze.
• Flushing the stomach and peritoneal cavity with warm sterile saline is an option. Use a gavage needle for increased pressure to flush the stomach, flushing contents onto gauze.
10. Use a clamp to ensure consistent removal of 80% of the stomach between mice. The clamp is placed at 0.5 cm from the lesser curvature which is the region between the esophagus and the duodenum. Clamp the stomach between the base of the esophagus and up to the pancreatic vessel. Micro Mosquito Forceps (RS-7117, Roboz Surgical Store, Gaithersburg, MD) is the preferred clamp with the curved jaws facing down (**Figure 5B**). Ideal positioning of the clamp allows for enough gastric tissue to be resected to allow for gastric transit, while not occluding the lower esophageal sphincter (LES) and gastroduodenal junction (GDJ). Occlusion of either or both of these structures will lead to the inability of food to pass through the stomach, ultimately leading to severe postoperative complications and death.
• Some groups may not use a clamp and prefer to use 6–0 Monocryl suture in a continuous suture pattern from the fundus to the pancreatic vessels to align where the cut will be (either absorbable or non-absorbable) (Garibay and Cummings, 2017)
11. Cut away the greater curvature of the stomach along the convex side of the clamp using spring scissors. Leave about 2 mm of stomach tissue extending from the clamp to allow for suturing. Ensure that no fundus remains. Use saline soaked cotton swabs to clean any additional food remaining onto the gauze.
12. Once the greater curvature of the stomach has been fully removed, use microneedle drivers to perform the continuous suturing technique using absorbable 8–0 Vicryl™ Violet braided sutures (J548G, 1x12” TG140–8 double armed, Ethicon, Raritan, NJ) along the clamp to close the approximately 2 mm of stomach extending from the clamp (**Figure 5C**).
13. Release the clamp and gently squeeze the stomach between two saline-soaked cotton swabs to check for any openings. Add additional sutures along the stomach if needed to ensure full closure. Check for openings along sutures.
14. If any bleeding occurs, press against the stomach suture line using two dry cotton swabs to encourage clotting for 20–30 seconds.
15. Surround the stomach (still external to the body) with more gauze to block the abdominal cavity and flush the stomach with the pre-warmed 0.9% saline in a 10 ml syringe with a 25-gauge needle. Keep the saline syringe warm by keeping it under a heating pad until needed.
16. Replace the stomach into the peritoneal cavity.
17. Use Pakistan needle drivers to close the muscle layer with a continuous suture pattern using 5–0 coated Vicryl™ Violet sutures (VCP391, 1x18” FS-2, Ethicon, Raritan, NJ).
18. Finally, use Pakistan needle drivers to close the skin layer with 2–3 interrupted sutures followed by 7 mm wound clips (RF7CS, Braintree Scientific, Braintree, MA) along the incision site.

### 2.5 Animal post-operative care

Note: the researcher is responsible for post-surgery care and monitoring as soon as the surgery has been completed until the mouse is fully recovered.

1. Immediately after surgery, place the cage on a 37°C heating pad (HotDog Veterinary Warming, Augustine Surgical Inc, Eden Prairie, MN) with 1/2 of the cage hanging off the heating pad and 1/2 of the cage seated on the heating pad overnight. Some may choose to leave mice on a heating pad (Harvard Apparatus Thermal Barrier, Holliston, MA) for up to 5 days. Mice should be carefully monitored as anesthesia wears off for potential complications. Mice should be awake within 5 minutes after surgery is completed and moderately ambulatory within 1 hour after surgery. Hunched posture is normal due to the incision, stitches, and staples; however, mice should be regularly monitored for distressed breathing or bleeding from the surgical site for the first 6 hours after surgery.
2. Bedding may be removed or kept in the cage. Both have been successful with regard to survival.
3. Inject 0.75 mL of warm sterile saline (0.9% NaCl) SQ over the shoulders of the mouse immediately following surgery to prevent dehydration (**Figure 5D** cartoon).
4. For 24 hours following surgery, make available 1 cup of DietGel (2 oz cup) with about 0.35 oz of Ensure Original Nutrition poured on top of the gel in addition to their solid diet. Their normal diet can also be placed back into the cage in the gel cup immediately after surgery or the next day. (**Figure 5D**). Researchers should ensure that mice are willingly eating solid food prior to removal of the gel cup. It may be necessary to leave food on the floor of the cage to encourage consumption if mice are showing signs of strain on their surgical site when reaching up to elevated food holders.
5. Monitor mice every 15 minutes immediately after surgery until they have regained the ability to ambulate.
6. Once ambulation has been achieved, which should happen quickly after a successful surgery, monitor them and the incision every 12 hours. Some picking at the staples is normal. However, if mice have removed the staples within the first 24 hours, they should be replaced. Incision site should be carefully examined for signs of internal bleeding which can be visualized through the skin at the shaved incision site. Internal bleeding may indicate the stomach sutures were not tight enough. In the event internal bleeding is observed, humane euthanasia should occur to prevent pain and suffering. Normal ambulation should be regained within 48 hours after surgery. Failure to regain full mobility indicates a complication and the mouse should be examined closely to ensure lack of internal bleeding. Supportive care should be provided, including softened solid food and the DietGel-Ensure liquid diet.
7. Administer Carprofen (10 mg/kg) 24-, 48-, and 72-hours post-surgery SQ over the shoulder area (**Figure 5D**).
8. Female mice can be grouped housed after surgery. Males that are littermates and grouped together before surgery may remain group housed. Single housing of mice should be considered if fighting or if quantification of food intake of individual mice is necessary, although the stress of single housing social animals will impact study outcomes and must be considered.
9. Over the course of postoperative care, monitor daily eating behavior, water intake, grooming, and general activity levels of the mice to watch for postoperative stress. If any abnormal behaviors are noted, contact your on-site Laboratory Animal Care Unit veterinarian on appropriate steps to mitigate health concerns.

## 3 Isolation of intestinal contents and Peyer’s patches protocol

The gut microbiome and gut immunity are components of cancer immunity (Almonte and Zitvogel, 2025). Obesity, cancer, and cancer therapies impact the gut microbiome (Almonte and Zitvogel, 2025; Pingili et al., 2021; Sipe et al., 2020). We have recently reviewed the role of FMT in cancer with extensive details on study design, from choice or murine model to mixing bedding, to endpoint studies (Bohm et al., 2024). Here, we present methods to isolate the intestinal contents of the cecum, akin to the human colon to provide a reliable method established by our experience and supported by our published works (Bohm et al., 2025, 2024; Leone et al., 2015; Martinez-Guryn et al., 2018; Pierre et al., 2019, 2023; Pingili et al., 2021; Xu et al., 2020). Makowski lab has published on the critical role that the post-bariatric surgery gut microbiome plays in cancer immunity by performing FMTs to demonstrate that the protection afforded by surgery such as VSG can be transplanted from one mouse to another (Bohm et al., 2025; Sipe et al., 2022). Further, we demonstrated similar protections in fecal transplants from patients with samples isolated before and after bariatric surgery (Bohm et al., 2025). In both murine and human fecal transplants, we demonstrated a role for the gut microbiome in boosting cancer immunotherapy, potentially through microbial derived metabolites and natural killer T (NKT) cells (Bohm et al., 2025). To aid other researchers in designing studies around obesity, cancer and the gut microbiome, the procedures are detailed here. While fecal pellets can be isolated from mice at baseline and throughout the study, the isolation of intestinal contents and Peyer’s patches is performed at endpoint. All supplies, reagents, and materials noted below are listed in **Table 3**. Ensure that aseptic technique is maintained when obtaining intestinal contents and scrapings and Peyer’s patches, with more detailed gut microbiome protocols in cancer studies as described in Bohm et *al.* (Bohm et al., 2024). All tools and the work surface must be disinfected with a facility-approved disinfectant (such as Peroxigard Ready-to-Use One Step Disinfectant Cleaner & Deodorizer or 70% ethanol). Between mouse samples, disinfect the work surface and tools. Use sterile autoclaved collection tubes.

**Table 3:** Supplies for intestinal contents and Peyer’s patches.

1. Isoflurane
2. Surgical Scissors
3. Spring Scissors
4. Forceps (fine tipped)
5. 1.5 mL Eppendorf Tubes
6. Petri Dish
7. Microscope Slide
8. Ice in large bucket

List of supplies and reagents needed to prepare for the isolation of fecal contents or tissue at endpoint.

### 3.1 Removal of the digestive tract

**Figure 6:**
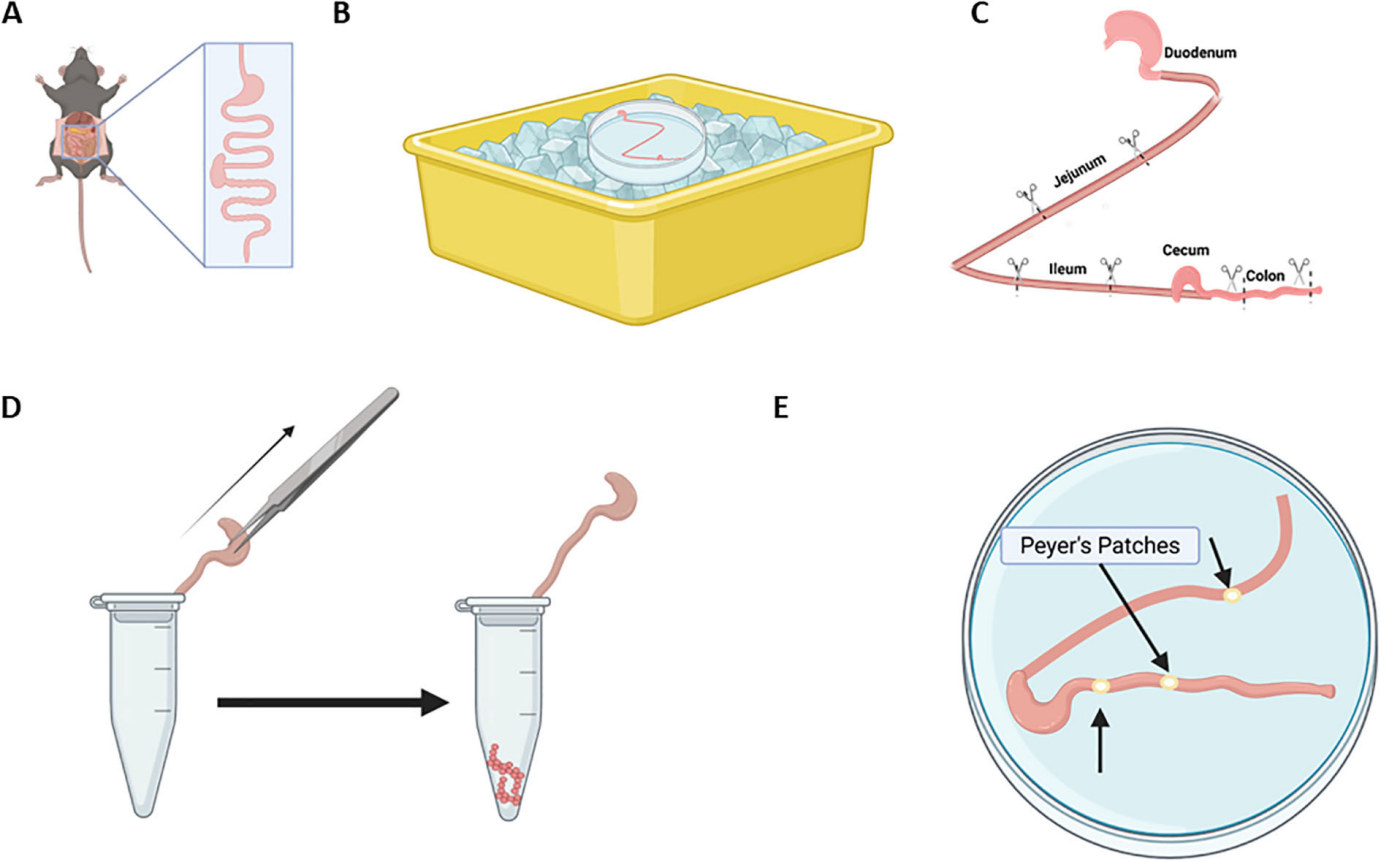
Digestive tract layout for isolation of intestine, fecal content, and Peyer’s patches. **(A)** Remove the intestinal tract from the esophageal-stomach juncture to the rectum. Figure is not drawn to scale. **(B-C)**. Lay the intestines in a petri dish sitting in a bucket of wet ice **(B)** in a “Z”-like pattern as shown in **(C)** from the stomach to the colon. Figure is not drawn to scale. **(C)** Cut 0.5-inch sections of the intestines from the duodenum, jejunum, ileum, cecum, and colon. **(D)** To collect luminal contents (fecal material) for microbiome examination, use fine tipped forceps to drape intestine section into a labeled tube. Gently close the lid but allow for the intestine to be pulled from the tube to extrude fecal contents. **(E)** To isolate immune cells along the intestine called Peyer’s Patches, identify the patches as small white bulges along the exterior wall of the intestine. Using small spring scissors, excise the patches, collect in a tube, and keep on wet ice for flow cytometric studies, or snap freeze in liquid nitrogen for subsequent histologic or RNA analysis.

1. Pre-label all collection tubes or cassettes for histology.
2. Euthanize mouse with isoflurane and perform a secondary method of euthanasia in accordance with IACUC protocol.
3. Using sharp surgical scissors, make a midline laparotomy incision to open up the skin, leaving the abdominal wall intact, from the base of the rib cage down to the lower abdomen (**Figure 6A**).
4. With closed scissors, separate the skin from the muscle layer.
5. Open the abdominal cavity, cutting from the xiphoid process down to the bottom of the digestive tract.
6. Using closed forceps, lift the liver to locate the stomach.
7. Identify where the esophagus terminates into the stomach and cut at that junction.
8. Gently grasp the stomach with forceps and lift the stomach out of the abdominal cavity.
9. Continue lifting the stomach, applying gentle pressure to unravel the small intestine from the mesenteric adipose tissue.
• Note: continuously slide the forceps down the small intestine while lifting, closer to the mouse cavity as more of the intestines are removed to minimize the chance of tearing.
10. Once the cecum reaches the band of mesenteric adipose, trim the adipose away from the intestines to release the cecum and colon.
11. Cut the pelvic bone to reveal the end of the colon and cut away from the rectum.
12. Once you have fully removed the digestive tract, place it on top of a dry petri dish that is embedded in ice (**Figure 6B**).

### 3.2 Collect intestinal contents and scrapings

1. Lay out digestive tract in a relative “Z” formation (**Figures 6B,C**) on the petri dish sitting in ice. There are no strict anatomical markers to divide up sections of the intestine (Uchida et al., 2013). In general, the small intestine length of an adult mouse is about 25–40 cm which includes the duodenum (connected to the stomach, about ~1–2 cm) to the jejunum (longest section, ~14–22 cm) to the ileum (ending at the cecum, ~10–16 cm). The cecum is a large pouch at the start of the colon (Bowcutt et al., 2014; Gu et al., 2013; Uchida et al., 2013). These measurements should be optimized for the age, strain, and disease status of your mouse model.
2. Begin by cutting approximately 0.5-inch (1.27 cm) sections from the center of the jejunum, ileum, and colon respectively and removing the entire cecum (**Figure 6C**).
• Note: remaining intestines can be set aside on another petri dish on ice for histology, isolation of Peyer’s patches, etc.
• Note: scissors must be very sharp to separate the jejunum from the ileum in a single cut, limiting the distal movement of intestinal contents that would occur if multiple cuts were used to separate the sections. Additionally, clamps or ligature may be used to prevent distal movement.
• Please note that if the animal has diarrhea or watery digesta due to planned experimental intervention, caution should be taken regarding regions of isolation along the intestine to avoid movement between sections, or exclude the animal due to sickness if no disease is anticipated.
3. To collect luminal contents:
• Use fine tipped forceps to drape an intestine section in an Eppendorf tube; do not release the section of intestine by maintaining hold of the forceps.
• Close the lid of the tube slightly, leaving enough of the intestine out of the tube so that the forceps can maintain their grip (**Figure 6D**).
• Hold the Eppendorf tube lid in place while gently pulling the intestine out of the tube so that the contents are extruded from the intestines and remain behind (**Figure 6D**).
• Close the lid firmly and snap freeze in liquid nitrogen.
• Repeat for remaining intestinal samples.
4. To obtain mucosal scrapings (microbes adhered to the intestine wall, which will also capture murine intestinal cells):
• Note: no scrapings are collected from the cecum.
• Inserting one blade of fine tipped, spring scissors into the opening of the intestinal section, leaving the other blade on the outside.
• Carefully cut through one side of the intestinal wall until the entire section is flayed open.
• Holding the section down with forceps, use a clean microscope slide, press down gently but firmly, and scrape down the inside of the intestinal section. One must verify that the scraping is done sufficiently to estimate the microbiome at the mucosal layer. Importantly, we have not compared scraping methods for the best outcomes as yet.
• Scrapings should be mostly collected on the edge of the slide, then scrape slide off material into an Eppendorf tube.
• Close and snap freeze in liquid nitrogen.

### 3.3 Isolation of Peyer’s patches

1. Lay out the lower small intestine on a petri dish on ice.
2. Identify the Peyer’s patches as small white bulges on the outside of the intestinal wall along the intestine (**Figure 6E**).
3. Using small spring scissors, excise the Peyer’s patches, removing as much of the intestinal wall as possible.
4. Place Peyer’s patches in an Eppendorf tube and keep on wet ice for flow cytometric studies, or snap freeze in liquid nitrogen for subsequent histologic, RNA, or other analysis

## 4 Discussion

Two-thirds of adults are obese or overweight, with increasing incidence in women, and disproportionately high incidence in minorities (Flegal et al., 2016). Lifestyle interventions including increasing physical activity and adhering to specific diets are first lines of interventions for obese or overweight patients. Specific diets may include low calorie, low carbohydrates, high protein, or high fruits and vegetables in combination with low red meat, such as the Mediterranean diet (Ruban et al., 2019). Significant weight loss can be achieved and maintained with diets like the Mediterranean when accompanied by physical activity (Dieli-Conwright et al., 2018; Look, 2014; Morey et al., 2009). However, diets and exercise often have low adherence rates, limiting their effectiveness over long periods of time (Demark-Wahnefried et al., 2012; Lemstra et al., 2016). Low socioeconomic status is also associated with higher rates of obesity due to a variety factors, including limited access to healthy food, less safe neighborhoods to exercise, lack of investment in the built environment (sidewalks, parks, etc.), among other factors which further exacerbate the risks of developing obesity and reduce the feasibility of lifestyle adaptations (French et al., 2019). Due to the difficulties of maintaining weight loss via lifestyle adaptations, bariatric surgery is an appealing alternative. Bariatric surgery reduces all-cause mortality by an average of 50% with a reduction in cardiovascular diseases, diabetes, and other pathologies (Kauppila et al., 2019).

The benefits of bariatric surgery on cancer risk and cancer outcomes have become increasingly clear in the past 5 years. Decreased risk of cancer has been reported in diverse patient cohorts following bariatric surgery compared to non-surgical controls, whether the cancers were obesity-associated or not (Adams et al., 2009; Aminian et al., 2022; Ashrafian et al., 2011; Bruno and Berger, 2020; Feigelson et al., 2020; Playdon et al., 2023; Schauer et al., 2017, 2019). In a 2020 study investigating cancer incidence, the risk of developing breast cancer was significantly reduced among obese women who had previous bariatric surgery (Feigelson et al., 2020). In fact, premenopausal obese women with highly aggressive estrogen receptor negative (ER-) breast cancer had the most pronounced reduction of breast cancer risk, compared to postmenopausal obese women with ER-positive (ER+) breast cancer after bariatric surgery (Feigelson et al., 2020). Furthermore, the weight loss effects of bariatric surgery have also proven to decrease medical expenditures (Ward et al., 2021). Despite many studies showing benefits in obesity-associated and even non-obesity-associated cancer, and the most aggressive cancer subtypes, the underlying mechanisms remain uncertain, which necessitates more research to understand this phenomenon.

Rodent models are a popular choice among researchers to study the effects of DIO (Bohm et al., 2022). Three mouse strains are commonly used for these studies—C57BL/6 (“B6”), FVB/N (“FVB”), and BALB/c—and each strain has variable susceptibility to DIO (Bohm et al., 2022). Numerous preclinical studies have implemented several types of bariatric surgeries in various rodent models. As B6 is the most susceptible to DIO, it is the preferred mouse strain compared to FVB and BALB/c strains, which are less obesogenic or resistant to DIO, respectively (Bohm et al., 2022). Yin et *al.* first described several surgical models used in DIO mice which demonstrated both short- and long-term effects on metabolism (Yin et al., 2011). Multiple models of bariatric or metabolic surgery were developed including gastric banding, RYGB (Liou et al., 2013), modified RYGB (mRYGB), biliopancreatic diversion, and VSG (Yin et al., 2011). Although all 5 surgical models produced significant weight and body fat loss, the VSG and mRYGB models were the most reliable and had lower complications and mortality rates compared to the other 3 types of surgeries (Yin et al., 2012, 2011). The enterogastro anastomosis surgery is a less-common procedure utilized by Amouyal et *al.* to examine type 2 diabetes after bariatric surgery in leptin-deficient *ob*/*ob* mice (Amouyal et al., 2020). Lastly, the bile diversion procedure has been used to examine dependence of FXR on obesity and glucose tolerance (Pierre et al., 2019). Our procedure of choice is the VSG because of its important role in patient care (Bohm et al., 2022). VSG is the first line of surgical intervention for bariatric surgery patients and is the most common bariatric procedure performed worldwide (Liou et al., 2013). Some studies have reported weight re-gain in mice after VSG, thus we believe that our methods described in this manuscript which demonstrates little weight rebound may be superior (Hao et al., 2017; Stevenson et al., 2021; Ye et al., 2020). However, in most publications, improved components of metabolic syndrome include sustained weight loss, improved glucose and insulin parameters, and increased browning of white fat are seen after bariatric surgery. Liou et al. (2013) examined alterations to the gut microbiota following RYGB-induced weight loss in mice and demonstrated that a fecal microbial transplant of gut microbes from mice post-RYGB into germ-free naïve mice was sufficient to cause weight loss in recipients. Fecal microbial transplants (FMTs) are a powerful investigative tool to isolate the effects of the gut microbiome in cancer risk or therapeutic outcomes in cancer, which was recently reviewed by our group (Bohm et al., 2024). We recently reported that bariatric surgery specific microbiome from both murine models and patients improved immunotherapy in a lean recipient pre-clinical model of breast cancer through FMT studies (Bohm et al., 2025). These studies have identified microbial derived metabolites branched chain amino acids as mediators of NKT cell activation and improved immunotherapy effectiveness through both observational and interventional (supplementation and *in vitro*) studies.

**Figure 7:**
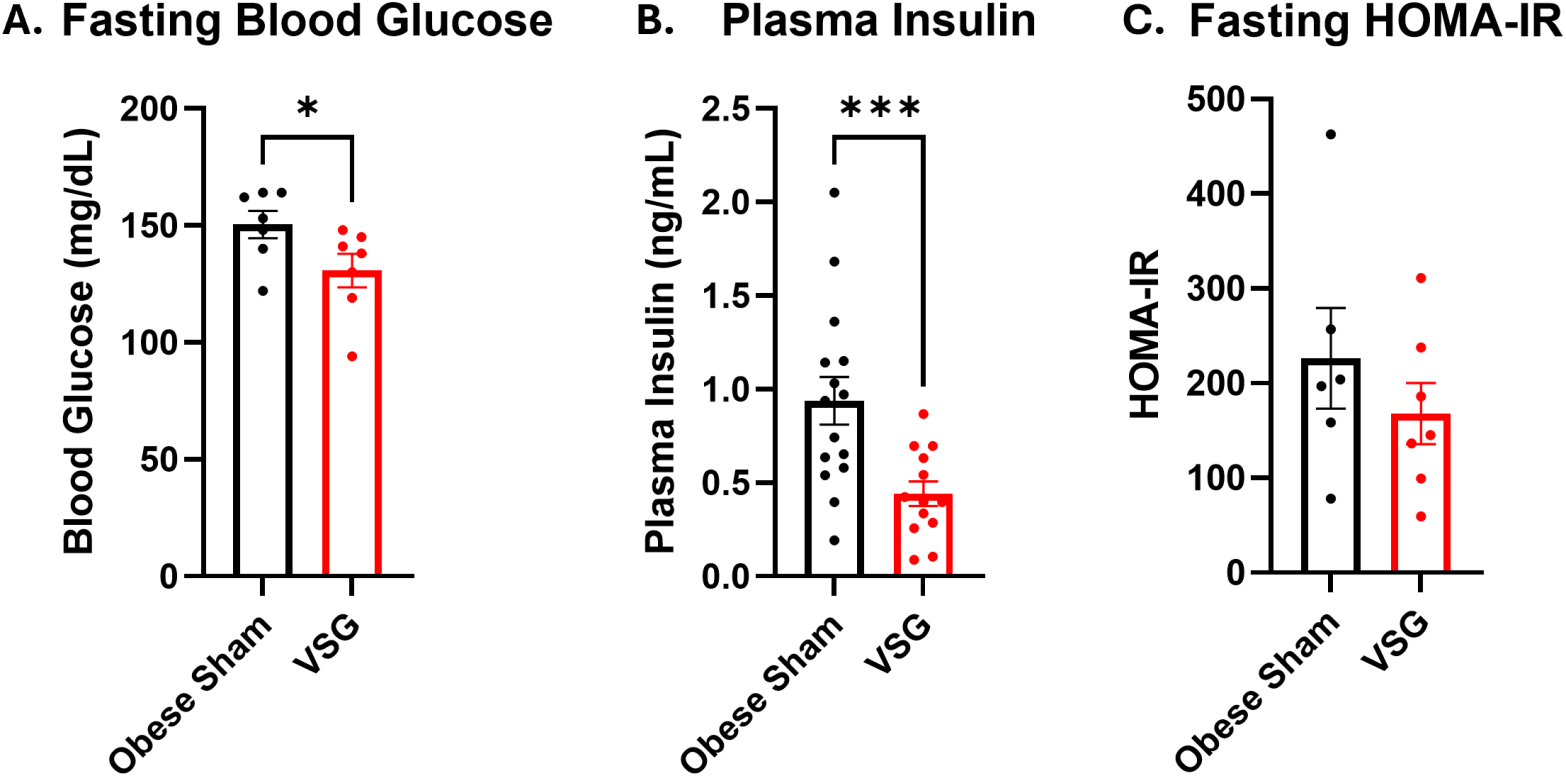
Anticipated outcomes for VSG surgery. **(A)** Blood glucose, **(B)** Insulin concentration in plasma, and **(C)** HOMA-IR score measured at endpoint after 4 hours of fasting. Endpoint occurred 5 weeks after VSG intervention. N = 12-15. Data presented as mean ± SEM with one-way ANOVA. *p<0.05, ***p<0.001.

In both our and other previously published reports, bariatric surgery, such as VSG, resulted in many improvements in metabolic outcomes. For example, in diet-induced obese B6 females, the Makowski lab demonstrated that mice lost on average 20% of their starting weight (12g) while being maintained on a high fat diet (Sipe et al., 2022). The weight loss began immediately after VSG and stabilized by 2–2.5 weeks post-surgery (Sipe et al., 2022). Weight regain was not observed in our Sipe et *al.* study, which is the basis of this methods approach detailed herein. Furthermore, in male C57BL/6 mice treated with various bariatric surgical approaches including VSG, RYGB, and bile diversion, Wasserman et al. demonstrated that mice lost approximately 30% body weight starting immediately after surgery and hitting a nadir at 3–4 weeks post-surgery, although weight rebound occurred in every surgical group (Yin et al., 2011). After bariatric surgery, examination of metabolic outcomes is important and spans from including quantification of fed and fasted blood glucose, to glucose and insulin tolerance tests or clamp studies, to body composition, fat pad mass, adipocyte size, crown like structures, local and circulating hormones, cytokines, chemokines, and other measures including cancer outcomes (Ding et al., 2016; Douros et al., 2018; Flynn et al., 2015; Garibay and Cummings, 2017; Hutch et al., 2021; Li et al., 2019; Sipe et al., 2022; Wang et al., 2018). The Pierre lab has published on the role of bile diversion surgery and changes to bile acids and gut microbiome in obese mice (Pierre et al., 2019). Previous literature in the field indicates that VSG will significantly lower fasting blood glucose levels and HOMA-IR score when compared to mice treated with sham surgery (Ding et al., 2016; Douros et al., 2018). Indeed, our VSG model significantly reduces both fasting blood glucose and plasma insulin levels at endpoint compared to obese sham controls (**Figures 7A, B**) though HOMA-IR was not significantly changed (**Figure 7C**). Many groups have published on obesity and weight loss with various outcomes associated with metabolic dysfunction which are reliable examples of metabolic measures (Bohm et al., 2025; Camp et al., 2023; Kong et al., 2024; Marathe et al., 2025; Pingili et al., 2021; Sipe et al., 2022; Zhao et al., 2024). Collection of intestinal contents to be used in subsequent fecal microbial transplant studies will enable clarification of the contribution of the gut microbiome to cancer risk and therapy after bariatric surgery (Bohm et al., 2024). Collection of fecal content by snap freezing in liquid nitrogen, or collection directly into sterile reduced (no oxygen) PBS with glycerol (15%) will allow viability of the microbes for subsequent FMT procedures (Bohm et al., 2025; Leone et al., 2015; Martinez-Guryn et al., 2018; Pierre et al., 2023). This is accomplished by leaving sterile PBS in an anaerobic chamber (0% oxygen, 5% hydrogen, 5% CO2, 90% nitrogen) for 72 hours to remove oxygen before adding sterile glycerol (Bohm et al., 2025, 2024). We have published using resuspension of cecal slurry at approximately 25 mg/mL prepared in glycerol/PBS under reduced oxygen anaerobic conditions (Bohm et al., 2025), with FMT recently reviewed in detail by our group (Bohm et al., 2024). Likewise, examination of Peyer’s patches and the immune cells within with flow cytometry or RNAseq will inform on local and systemic impacts of bariatric surgery on immunity. Previous gut sampling approaches have been described (Chitrala et al., 2017; Dillenburg-Pilla et al., 2016; Katzberg and Morris, 1987; Koester et al., 2021; Tong et al., 2014). Our method described herein is rapid and requires minimal buffers or reagents which is cost effective and therefore a potentially superior method. This will help improve viability for fecal microbial transplants and sequencing results, as demonstrated previously by our team (Bohm et al., 2025).

To specifically examine the protective impacts of bariatric surgery on the ER- breast cancer subtype, our group developed this VSG protocol to study the impacts of weight loss by VSG compared to various controls (Sipe et al., 2022). Female mice were fed a 60% kcal from fat high fat diet from Research Diets, Inc. (New Brunswick, NJ) starting at 4 weeks of age, with a 10% kcal from fat low fat diet control. After 16 weeks of diet, at 5 months of age, mice were subjected to VSG or sham surgeries. An important control group was weight matched through caloric restriction and received sham surgery. We reported that after VSG mice lost adiposity by EchoMRI analysis and demonstrated reduced gonadal and mammary fat pad mass and adipocyte size. Moreover, the mice improved other metabolic and obesity-associated chemokine and cytokine parameters which together resulted in significantly *reduced* breast cancer tumor burden (Sipe et al., 2022). Interestingly, despite most obesity-associated metabolic parameters improving after bariatric surgery, we discovered a VSG-specific increase in circulating IL-6; and showed that IL-6 induced checkpoint ligand PD-L1 on cancer cells (Sipe et al., 2022). This finding led to subsequent studies demonstrating that after VSG-induced weight loss, the response to immunotherapy was surprisingly improved in mice that received bariatric surgery and anti-PD-L1 therapy. Additionally, breast cancer progression was reduced compared to obese sham controls where immunotherapy was ineffective (Sipe et al., 2022).

An example of the type of impactful studies possible with the bariatric surgery studies described herein include the identification of a conserved adipose-specific transcriptomic gene signature between mice and humans. Through the comparison of transcriptomic changes in adipose tissue after bariatric surgery from both patients and mouse models a conserved bariatric surgery associated weight loss signature (BSAS) was identified (Sipe et al., 2022). Specifically, we examined adipose tissue from mice after receiving sham or VSG surgery and compared that to existing publicly available data from humans before and after bariatric surgery (Poitou et al., 2015). Importantly the BSAS significantly associated with decreased tumor volume (Sipe et al., 2022). Between human and mouse, 54 differentially expressed genes were identified in common associated with weight loss from bariatric surgery. We compared the BSAS with tumor volumes and found that 11 genes were significantly associated with tumor volumes which we termed tumor (T-BSAS). Excitingly, the T-BSAS gene signature resembled the tumors from lean mice fed low fat diet (Sipe et al., 2022). Importantly, Camp et al. demonstrated that VSG-associated weight loss led to epigenetic reprogramming in mice with VSG or dietary weight loss (Camp et al., 2023). Thus, bariatric surgery is a powerful tool to reduce weight in pre-clinical models, reduce breast cancer burden, improve immunotherapy effectiveness and will likely be used more often in future cancer studies.

Similarly to patients, VSG leads to few complications in mice. Acute complications from bariatric surgery or sham control surgery include failure to survive the surgery or death within 1–2 days due to several possibilities: not arising from isoflurane, poor cauterization and excessive bleeding, dehiscing (undoing) of the gastrectomy stitches due to poor technique, or the animal or other animals interfering with the closure stitches. For the latter reason, all mice in a cage must undergo surgery, not just a select few, so that no mouse is targeted during recovery. Regarding acute surgery-associated issues, we ensure that all surgeons are well trained with extra time spent learning specific techniques from the veterinary staff, including certification in aseptic technique and a workshop on sutures. We encourage all staff and trainees to practice suturing before training with the veterinarians. It should be stressed that while sutures are expensive, training with or using expired sutures is not recommended because of excessive breaking. If the methods and techniques defined here are followed, in close consultation with the veterinary staff, once trained, laboratory staff, students and fellows should have ample success. Additionally, if a mouse does not survive surgery, it is important to perform a dissection with the veterinary staff to determine cause of death and identify techniques that may be improved. The VSG procedure defined in this method resulted in 100% survival in B6 females with no weight regain out to 5 weeks after surgery. Long term complications may arise in three ways. First, weight regain due to regrowth of the stomach is quite common in some publications (Garibay and Cummings, 2017; Yin et al., 2011), although in work by our group and in other publications weight rebound was not observed (Hutch et al., 2021; Sipe et al., 2022). While the mouse may regain weight due to stomach re-expanding - possibly due to not enough of the stomach being removed - the mice typically do not regain up to the body weights of sham controls, which affords continued benefits of bariatric surgery. It is not clear why some mice regain weight while others do not, which is why a standardized method such as reported herein will help the field be able to report across studies. Second, a complication after surgery is that adhesions may occur in the peritoneal cavity. While adhesions typically do not impair the quality of life of the mouse, it makes dissection of certain organs as endpoint more intensive than in a mouse that did not have surgery. Lastly, a third complication at the time of death or, if extreme, a cause of death, a bezoar may be present in the stomach. A bezoar is a mass of food, bedding, or hair that accumulates in the stomach. Why material accumulates in the stomach is unclear. It is possible that the stomach stretches post-surgery and the material in the stomach does not digest well due to a weak stomach wall or other defects. Alternatively, defects in digestion may lead to the food accumulating and the subsequent stretching of the stomach. Most bezoars are small and discovered at endpoint, but an occasional bezoar can be large at over 2–3 cm, tightly compacted, or containing purulent material, which will impair the quality of life and lead to an early IACUC determined endpoint. In our hands, bezoars rarely occur in a small subset of mice in the B6 strain, 1–5% of mice after VSG, while in the FVB/N strain it is estimated to be at about 20%. In studies conducted by our group using the FVB strain, survival was overall very poor after VSG; approximately fifty percent of the mice died about 48 hours after VSG, with the other 50% dying after about 2 weeks. The FVB mice that lived past the acute recovery stage developed large bezoars which were associated with inhibited digestion. These results from our lab suggest that although surgery can be performed the same in each strain, the background strain is relevant to survival after bariatric surgery, although these findings must be further examined. It is unclear how bezoars form or how to prevent them, as they are rarely reported as complications in the literature.

## 5 Conclusion

In conclusion, bariatric surgery results in sustainable weight loss over a lengthy period compared to lifestyle interventions and has proven to be protective in both obesity-associated and obesity-independent cancer risk. Of note, the use of medical weight loss pharmaceuticals has skyrocketed in recent years with the development of incretin memetics and GLP-1 receptor agonists. However, each patient’s eligibility varies depending on their physical characteristics, such as BMI, and use may be limited by availability of the drugs, ability to pay out of pocket, insurance coverage, or side effects which may limit the widespread use of these drugs (Davies et al., 2015; Grunstein et al., 2023; Ruban et al., 2019; Sahebkar et al., 2017; Sonoda et al., 2008; Torgerson et al., 2004; Wilding et al., 2021). The role of pharmaceuticals such as semaglutide and other GLP-1 receptor agonists is of great interest because it is unknown if medical weight loss will achieve the same cancer-preventive benefits as observed after weight loss by bariatric surgery. We have published that in murine models, weight loss by GLP-1 receptor agonist semaglutide (Ozempic or Wegovy) compared to a novel triple receptor agonist retatrutide reduced both tumor onset and progression in pancreatic and lung cancer (Marathe et al., 2025). Even after retatrutide was discontinued and weight was regained to baseline levels, protection from tumor growth was persistent (Marathe et al., 2025). These exciting findings point to a role for medical weight loss as cancer protective but must be studied in patients in the future. Likewise, nutraceutical approaches such as probiotics, vinegar, and herbs are possible to impact the gut microbiome in obesity and fatty liver to affect cancer onset or growth (Cao et al., 2022; Liu et al., 2025; Newman et al., 2023). The impact of VSG to improve anti-tumor immune response after anti-PD-L1 was profound in our pre-clinical studies (Sipe et al., 2022), however, it remains unclear if improved response to immunotherapy will also be found in patients after bariatric surgery. In fact, the immunosuppression associated with obesity actually leads to improved response to immunotherapy in some cancers such as melanoma, renal cell carcinoma, and non-small cell lung cancer, but it is unclear of the impacts on breast cancer (Woodall et al., 2020). Therefore, future studies are necessary in former bariatric surgery patients receiving immunotherapy treatment for their cancer. Establishing a clear and concise protocol for pre-clinical models for bariatric surgery will benefit the cancer field and provide necessary rigor and reproducibility, especially a protocol established to be successful in female mice, which are rarely studied in the literature. Herein, we have a detailed protocol to perform the VSG and sham controls in a murine model, with collection of fecal content and immune cells, which will aid in advancing the study of bariatric or metabolic surgery to determine impacts on cancer.

## Data Availability

The original contributions presented in the study are included in the article/supplementary material. Further inquiries can be directed to the corresponding authors.
